# Temporal and spatial characteristics of bone conduction as non-invasive haptic sensory feedback for upper-limb prosthesis

**DOI:** 10.3389/fnins.2023.1113009

**Published:** 2023-03-28

**Authors:** Raphael M. Mayer, Alireza Mohammadi, Ying Tan, Gursel Alici, Peter Choong, Denny Oetomo

**Affiliations:** ^1^Department of Mechanical Engineering, The University of Melbourne, Parkville, VIC, Australia; ^2^School of Mechanical, Materials, Mechatronic and Biomedical Engineering, University of Wollongong, Wollongong, NSW, Australia; ^3^ARC Centre of Excellence for Electromaterials Science, Wollongong, NSW, Australia; ^4^Department of Surgery, St Vincent's Hospital, The University of Melbourne, Parkville, VIC, Australia

**Keywords:** neuroprosthesis, sensory feedback restoration, human-robot interaction (HRI), tactile feedback, bone conduction (BC)

## Abstract

Bone conduction is a promising haptic feedback modality for upper-limb prosthesis users, however, its potential and characteristics as a non-invasive feedback modality have not been thoroughly investigated. This study aimed to establish the temporal and spatial characteristics of non-invasive bone conduction as a sensory feedback interface for upper-limb prostheses. Psychometric human-subject experiments were conducted on three bony landmarks of the elbow, with a vibrotactile transducer affixed to each to provide the stimulus. The study characterized the temporal domain by testing perception threshold and resolution in amplitude and frequency. The spatial domain was evaluated by assessing the ability of subjects to detect the number of simultaneous active stimulation sites. The experiment was conducted with ten able-bodied subjects and compared to two subjects with trans-radial amputation. The psychometric evaluation of the proposed non-invasive bone conduction feedback showed results comparable to invasive methods. The experimental results demonstrated similar amplitude and frequency resolution of the interface for all three stimulation sites for both able-bodied subjects and subjects with trans-radial amputation, highlighting its potential as a non-invasive feedback modality for upper-limb prostheses.

## 1. Introduction

Haptic sensory feedback plays an important role in effective closed-loop control of upper-limb prostheses (Saunders and Vijayakumar, 2011; Antfolk et al., 2013; Markovic et al., 2018; Stephens-Fripp et al., 2018; Farina et al., 2021), promoting the body ownership of prosthetic arm users (Canzoneri et al., 2013; Shehata et al., 2020; Richard et al., 2021) and the reduction of phantom limb pain management (Dietrich et al., 2012). This feedback is generated through a form of stimulation that is encoded with relevant feedback information and delivered to the user's residual limb. The sensory feedback information can be used in a variety of applications including upper-limb prosthesis, e.g., for grasp force control (Childress, 1980; Westling and Johansson, 1984; Augurelle et al., 2003; Antfolk et al., 2013), haptic applications e.g., robotic teleoperation (Dahiya et al., 2010) or virtual reality applications (Richard et al., 2021).

Invasive and non-invasive haptic sensory feedback approaches have been investigated in the past (Cordella et al., 2016; Stephens-Fripp et al., 2018). Invasive approaches, such as implanted nerve electrodes, show great potential but limited applicability or desirability to a subset of the people living with limb loss due to the inherent surgical risks and potential limited lifetime of the electrodes (Schofield et al., 2014; Cordella et al., 2016; Farina and Amsüss, 2016; Svensson et al., 2017). Non-invasive stimulation will therefore continue to play a strong role in prosthetic applications (Farina and Amsüss, 2016). It is also directly applicable in other areas such as in haptics and human robot interaction (Goodrich and Schultz, 2007) and more generally in human machine interfaces (HMI) (Tahir et al., 2018). The state of the arts of non-invasive tactile feedback are conventionally applied on the skin through electrotactile, vibrotactile and mechanotactile modalities to varying degrees of success (Antfolk et al., 2013; Schofield et al., 2014; Sensinger and Dosen, 2020; Farina et al., 2021). The shortcomings of these methods were comprehensively studied and summarized in two thorough review papers (Svensson et al., 2017; Stephens-Fripp et al., 2018). These shortcomings include: (1) The force dependency of perceived sensation in vibrotactile feedback, which can affect the accuracy and consistency of feedback information when the transducer is pressed against the skin; (2) Changes in the perception of both electrotactile and vibrotactile stimulation on the skin with varying locations, making it difficult to achieve precise and reliable feedback; (3) The bulky and high power consumption setup of the mechanotactile feedback, which can limit its practicality in real-world applications.

This paper focuses on the investigation of a non-invasive bone conduction modality as an interface to convey information to the human user. Bone conduction is a method of providing vibrotactile feedback through the bone. This approach relies on the transmission of vibrations/sound through the bone, which stimulates the Pacinian Corpuscles located around the bone (Clemente et al., 2017). Bone conduction is a relatively recent and emerging modality which can potentially address the aforementioned shortcomings.

The potential of bone conduction as a haptic feedback interface for upper-limb prosthesis has been studied in Clemente et al. (2017) through mechanical stimulation of a bone-anchored (osseointegrated) prosthesis. Superior bandwidth compared to vibrotactile feedback on the skin was found in Clemente et al. (2017) for invasive bone conduction. This allows for richer feedback of sensory information to the human user.

While the results of the osseoperception using bone conduction were promising, it is only applicable for users with osseointegrated upper-limb prostheses. There are cases that osseointegration is not suited for people with upper-limb loss due to the lack of length or strength in the residual limb or the inherent surgical risks of the invasive technique (Schofield et al., 2014; Cordella et al., 2016; Farina and Amsüss, 2016; Svensson et al., 2017). Therefore, non-invasive options such as stump sockets are expected to continue to play an important role in prosthesis.

In Mayer et al. (2019), the authors proposed the non-invasive bone conduction as haptic feedback system through vibrotactile stimulation of bony landmarks of the elbow. The preliminary results, with limited number of psychometric parameters and subjects, demonstrated comparable performance to the invasive bone conduction method (Mayer et al., 2019, 2020c). It provides a higher sensitivity of the perception of lower frequencies, allowing for the use of lower stimulation forces and therefore smaller and lower power consuming transducers. In addition, it has been observed that the non-invasive bone conduction is independent from the force pressing the transducers against the human subject (Mayer et al., 2018). This is an important characteristic as volume fluctuations, present in residual limbs (Sanders et al., 2012; Paterno et al., 2018), no longer affect the perception of the provided sensory feedback.

The objective of this paper is to thoroughly investigate and determine the temporal and spatial characteristics of the non-invasive bone conduction with different user groups including subjects with amputation. The temporal parameters of the interface will be characterized by the lowest perceivable stimulation threshold and the smallest perceivable resolution in amplitude as well as frequency. The spatial parameters define the capabilities of the interface to perceive stimulation on multiple sites on the physiologically given bony landmarks on the elbow when stimulation was applied one-at-a-time. This is of interest in prosthetic grasping as combination of different types of feedback information are required (Westling and Johansson, 1984; Johansson and Westling, 1987; Augurelle et al., 2003; Mayer et al., 2020b). The temporal and spatial characteristics of the bone conduction has been conducted on both able-bodied subjects and subjects with trans-radial amputation and compared to each other and to the invasive bone conduction method.

## 2. Methodology

In this section, the measuring parameters, experimental setup, and protocol used to obtain temporal and spatial parameters as well as the statistical analysis are presented. The experiment was conducted with ten able-bodied subjects (SA) and two subjects with trans-radial amputation (ST), see Table 1. All subjects read the plain language statement and signed the consent form approved by the Ethics Committee of the University of Melbourne (Ethics Id 1852875.1).

**Table 1:** Subjects were in the following results of able-bodied subjects are indicated as (SA) and for subjects with trans-radial Amputation as (ST).

**Subject group**	**Number**	**Age**	**Gender**
		**(years)**	
Able bodied Subjects (SA)	10	26 ± 3.9	2 F, 8 M
Subjects with trans-radial Amputation (ST)	2	29 ± 8.5	0 F, 2 M

F, female; M, male.

### 2.1. Temporal and spatial parameters

The temporal domain is characterized by the perception threshold (PT) and the minimum noticeable difference for subjects, referred to as “just noticeable difference” (JND). The JND is obtained to quantify the perceivable resolution of the bone conduction interface for frequency and amplitude. The spatial domain is characterized by the ability to identify different stimulation sites (SPLIR).

#### 2.1.1. Perception threshold

PT is the minimum stimulation amplitude that subjects can perceive at a certain stimulation frequency at a certain stimulation site and can be represented as *PT*(*f, l*). For any given frequency *f* and site *l*, the amplitude thresholds changes for each person, as shown in Clemente et al. (2017, 2016), and Mayer et al. (2019), thus it is necessary to be identified.

#### 2.1.2. Just noticeable difference

The JND is determined for amplitude as well as frequency describing the resolution of the interface and therefore specifying the possible information rate of the interface. It is the minimum difference a subject can discriminate with a pre-determined probability. The *JND*_*f*_(*f, a, l*) varies with stimulation amplitude *a* for a given stimulation frequency *f* and site *l*. The *JND*_*a*_(*f, a, l*) varies with stimulation frequency *f* for a given stimulation amplitude *a* and site *l*. For a non-invasive bone conduction interface applied to the elbow (a natural location for the case of a transradial prosthetic arm), three accessible bony landmarks exist, namely the epicondylus (medialis and lateralis) and the ulnar olecranon. Therefore, multiple vibrotactile transducers can be deployed and potentially be used simultaneously. The spatial domain is therefore characterized by the spatial parameter single-point location identification rate (SPLIR).

#### 2.1.3. Single-point location identification rate

SPLIR is the success rate that the subject correctly identifies the correct stimulation site. SPLIR is different for each site *l*:


(1) $$\begin{array}{l} SPLIR\left(l\right)=\frac{N_{corr}\left(l\right)}{N\left(l\right)}, \end{array}$$

where *N*_*corr*_(*l*) is the number of correct identified stimulations for the number of presented stimulations *N*(*l*) at site *l*.

### 2.2. Experimental setup

**Figure 1:**
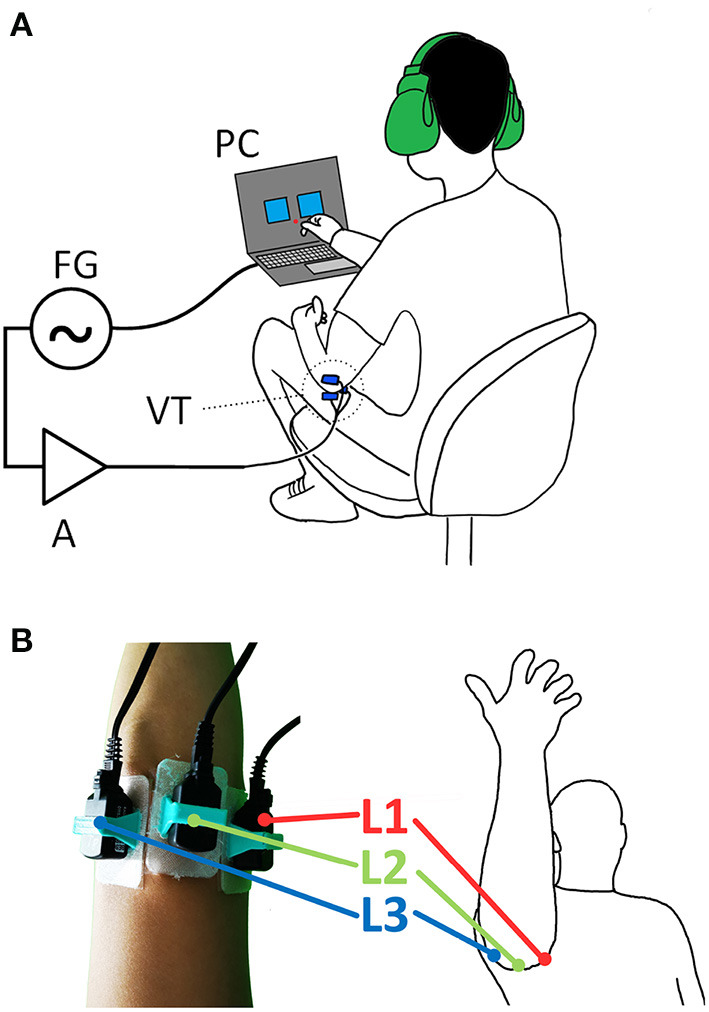
The experimental setup: **(A)** three vibrotactile transducers (VT) are controlled *via* a personal computer (PC) connected *via* USB to a frequency generator (FG) and an amplifier (A); **(B)** the transducers are fixed onto the bony landmarks (figure shows right arm) of the dominant hand: (L1) epicondylis medialis, (L2) ulnar olecranon and (L3) epicondylus lateralis in 3D printed holder and medical grade double sided sticker and mounted.

The experiment was conducted using the setup shown in Figure 1, where three *Vibrotactile Transducers (VT)* were driven by the *frequency generator (FG)* and *amplifier (A)* and controlled by the *personal computer (PC)*. The three transducers T1-T3 were placed onto the 3 bony landmarks of the elbow: epicondylis medialis (L1), ulnar olecranon (L2) and epicondylus lateralis (L3), see Figure 1.

#### 2.2.1. Vibrotactile transducers

Three B81 transducers T1-T3 from RadioEar Corporation (USA) were utilized to provide the vibrotactile stimulus to the bony landmarks. All three transducers were calibrated using an Artificial Mastoid Type 4930 from Brüel & Kjære (Denmark) adjusted to produce the same force output of 121.5 dB at *f* = 1 kHz. The transducers were affixed to the bony landmarks, see Figure 1, of the subjects using a 3D printed holder (PLA/TPU) and medical grade double sided sticker Type 1510 (3M™).

#### 2.2.2. Frequency generator

A National Instruments NI USB-6343 is used.

#### 2.2.3. Amplifier

A 15W Public Address amplifier type A4017 from Redback Inc. (Australia) with 4 − 16Ω output to drive the 12.5Ω B81 transducers and achieve a harmonic distortion of < 3% at 1 kHz.

#### 2.2.4. Personal computer

A Windows Surface Book 2 (Intel Core i7-8, 16GB RAM, Windows 10™) and a MATLAB^®^ GUI, guiding the user through the experiment and controlling the stimulation parameters.

### 2.3. Experimental protocol

The experiment is divided into temporal and spatial parameters. In order to reduce time effort for the subjects with trans-radial amputation, the able-bodied subjects carried out the whole experiment first. The longest time effort in the experiment was in the identification of the just noticeable difference. Therefore, this was carried out in stages: it was first done on the able-bodied subjects. The site with the lowest perception threshold was identified as L1. For the subjects with transradial amputation, this experiment was done for L1 instead of for all three sites, thus reducing the time-effort required from the subjects with amputation. The following explains the protocol for determining each parameter.

The temporal parameters, PT and JND, are obtained utilizing a single interval adjustment matrix (SIAM) method in order to reduce long trial times, requiring half the amount of repetitions compared to a standard two-interval forced-choice (2IFC). SIAM methods has been previously been implemented by Dosen et al. (2016) and shown to achieve same precision as a 2IFC test in Kaernbach (1990). In the SIAM procedure the outcome (hit, miss, false alarm, correct rejection) is used to adjust the signal level in a staircase manner. The response criterion is set to 0.5 which means the obtained PTs / JNDs are recognized with a 50% probability which is the same performance as in 2IFC tests (Kaernbach, 1990). This was chosen according to Kaernbach (1990) where it was shown that it results in the best threshold estimate.

A SIAM matrix,


(2) $$\begin{array}{l} SIAM=\left[\begin{array}{cc} -1 & 1\\ 2 & 0 \end{array}\right] \end{array}$$

as shown in Kaernbach (1990), achieves the best threshold estimate *via* a 50% target performance, i.e., half of the presented stimuli are blank. Blank stimulus means that it carries no stimulation for perception threshold and no change for JND tests. The provided stimulus *S*_*i*_ is therefore adjusted based on the response of the subject


(3) $$\begin{array}{l} S_{i+1}=S_{i}+SIAM\left(a,b\right)\delta S \end{array}$$

where δ*S* is the step size. The index *a* determines if the stimulus was a blank (*a* = 1) or a true stimulation (*a* = 0). The index *b* is determined by the subject's yes/no response, where a yes means *b* = 1 and a no means *b* = 0. A correctly perceived stimulation changes the stimulus by −1δ*S* while an incorrectly perceived stimulation increases the stimulus by +1δ*S*. An incorrect perception of a blank stimulation increases the stimulus by +2δ*S* while a correct perception of a blank keeps it at the current level (Kaernbach, 1990; Dosen et al., 2016).

#### 2.3.1. Perception threshold

**Figure 2:**
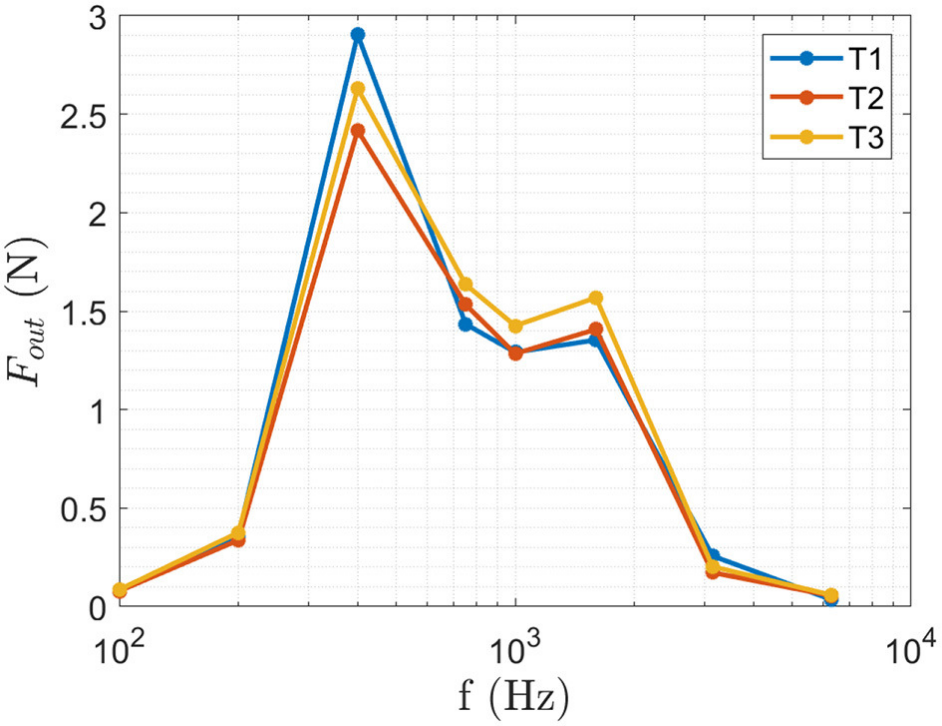
The calibration curve for the transducers T1-T3 is used to calculate the perception threshold in Newton from the recorded Voltage applied to the transducers. The produced force *F*_*out*_ was measured for *f* ∈ [100, 200, 400, 750, 1,500, 3,000, and 6,000] Hz at an amplitude of *a* ∈ [0.1, 0.3, 0.5] V where the figure shows the obtained results at *a* = 0.5 V.

A complete psychophysical evaluation of the perception threshold would imply determining the PT at a step size determined by the Just Noticeable Difference Frequency difference (JND_*f*_). As the JND_*f*_ is not known *a priori*, the preliminary results obtained in Mayer et al. (2018, 2019, 2020c) are used to define the frequency range, where the three sites L1-3 are each individually perceivable as well as dominant tactile perception is shown in a frequency range of *f* ∈ [100, 400, 750] Hz. The perception threshold *PT*(*f, l*) is obtained *via* SIAM (Kaernbach, 1990) method. The threshold for each frequency *f* at the site *l* is obtained by presenting 26 repetitions and the amplitude adjusted according to the subjects feedback *via* SIAM method. The frequencies *f* are presented in a randomized manner at each site *l*. To allow for a technical implementation and selection of suitable transducers the Perception Threshold is given in Newton utilizing the previously obtained calibration (Figure 2).

#### 2.3.2. Just noticeable difference

Similar to PT, a complete psychophysical characterization of the JND would imply determining a full range in amplitude/frequency (AF). This would result in an impractically large number of measurements, resulting in an excessively long duration of experiments for subjects. Hence, the AF domain was divided into discrete steps, called AF reference points, where *f*_*ref*_ ∈ [100, 400, 750] Hz and *a*_*ref*_ ∈ [0.1, 0.3, 0.5] V resulting in a combination of 9 AF points where the reference amplitudes and frequencies where chosen according to the preliminary results obtained in Mayer et al. (2019). At each AF reference point, the JND_*f*_ and JND_*a*_ were obtained in increasing direction (toward the maximum value). The JND is given in Volt, which can be used to derive the smallest necessary step size for an implementation and selection of suitable driver circuitry.

#### 2.3.3. Single-point location identification rate

Spatial parameters were obtained according to Mayer et al. (2020c). The subjects were asked to report on the site L1-L3 of the stimulation. Therefore, the subjects were presented with the stimuli on the three different sites without *a priori* knowledge of the stimulation site. The order of stimulation sites were applied randomly from *f* ∈ [100, 200, 400, 750, 1,500, 3,000, and 6,000] Hz, *a* = 0.5 V and each repeated 10 times. Frequencies are choose according to the shown bandwidth in Clemente et al. (2017) and Mayer et al. (2019). Each stimulation was ON for 1 s. At the start of the experiment, the subjects were provided with the opportunity to familiarize themselves with the stimulation and therefore explore the association of the three stimulation sites by voluntary inducing stimuli on each site.

### 2.4. Analysis

The statistical analysis in this study is utilized to investigate the difference between the physiological sites and the different subject groups.

#### 2.4.1. Effect of physiological locations

A non-parametric statistical analysis, specifically a Friedman Test (Daniel, 1990) was applied to compare the three physiological sites for each temporal and spatial parameter. In case of statistical significant differences, this was followed up by a *post-hoc* analysis *via* Wilcoxon signed rank test (Wilcoxon, 1945) to determine which of the three physiological sites was different. The *p*-value results are presented for the Friedman as well as the applied *post-hoc* Wilcoxon signed rank test.

#### 2.4.2. Perception threshold

The achieved results of perception threshold are visually presented by plotting the mean and the stand deviation as an error bar.

#### 2.4.3. Just noticeable difference

A summary plot shows the JND_*a*_ and JND_*f*_ at each AF reference point by its mean value, with the origin at the AF reference point. More details are shown in individual plots for each AF reference point for each site showing the obtained mean value and the stand deviation as an error bar.

#### 2.4.4. Single-point location identification rate

The achieved results of perception threshold is visually presented similar as in Mayer et al. (2020c) by plotting the mean and the stand deviation as an error bar.

#### 2.4.5. Difference between subject groups

A qualitative comparison between able-bodied subjects and subjects with trans-radial amputation (ST) was carried out to compare the two subject groups for each temporal and spatial parameter. The obtained mean values for the parameters are use for such comparison.

## 3. Results

In this section, the temporal and spatial parameters are presented and compared statistically for the different sites/bony landmarks (L1-L3) at the elbow and qualitatively across different subject groups.

### 3.1. Physiological sites

#### 3.1.1. Perception threshold

**Figure 3:**
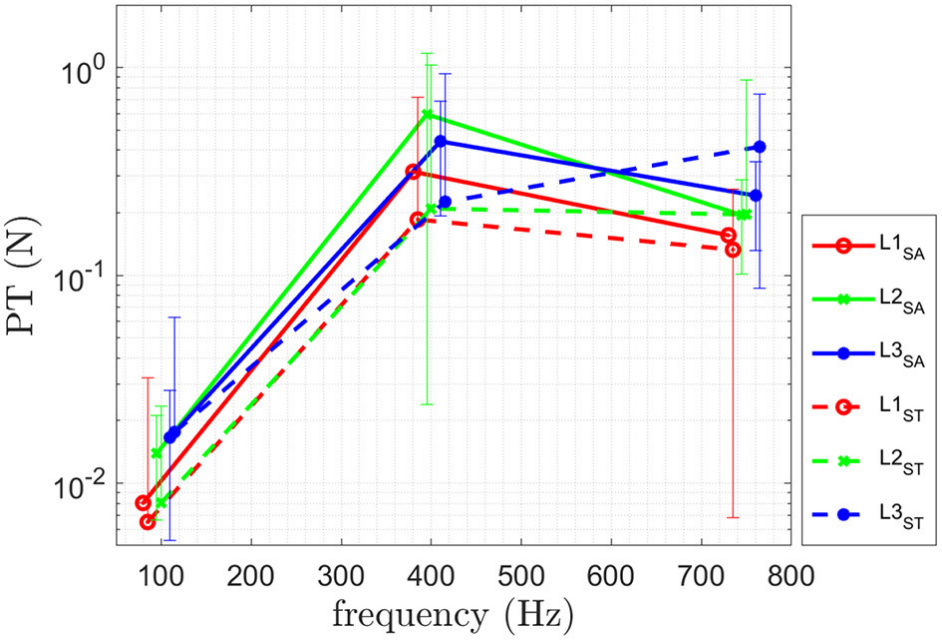
Results of the psychophysical evaluation for PT of ten able-bodied subjects (SA) and for two trans-radial amputees (ST). Means and standard deviations of the identified PT at frequencies [100 400 750] Hz are shown.

The results for PT are shown in Figure 3 and Table 2. The results of the obtained perception threshold, for the three physiological sites of able-bodied subjects (SA) and trans-radial amputees (ST) are shown in Figure 3. The mean perception thresholds for *SA* is [0.015 0.45 0.2] N and for *ST* [0.015 0.21 0.25] N for [100 400 750] Hz.

**Table 2:** The *p*-values of the Friedman for perception threshold comparing the three different physiological sites L1, L2, and L3 at all frequencies is shown.

		**Frequency**		
		**100 Hz**	**400 Hz**	**750 Hz**
Friedman	*L*1 vs. *L*2 vs. *L*3	≤0.001*	0.273	≤0.001*
Wilcoxon	*L*1 vs. *L*2 *L*1 vs. *L*3 *L*2 vs. *L*3	0.002* 0.002* 0.059	- - -	0.002* 0.002* 0.059

Significance level is *p* < 0.05. The *p*-values of the *post-hoc* Wilcoxon signed rank test for Perception Threshold comparing the three different physiological sites L1, L2 and L3 to each other over *f* ∈ [100, 400, 750] Hz of the ten able-bodied subjects is shown. Significance level is *p* < 0.05. *Indicates statistically significance.

The Friedman test results, comparing the three different sites, are shown in Table 2. The obtained results indicate a statistically significant difference in performance for [100 750] Hz for the perception threshold for the three different sites. No statistical difference is observed at 400 Hz. A *post-hoc* test (Wilcoxon signed rank test), is performed for [100 750] Hz and the corresponding *p*-values are shown in Table 2. In the following, the obtained results are summarized:

**L1 vs. L2:** A statistical significant difference for perception threshold at [100 750] Hz is shown in Table 2 with L1 having a lower PT.

**L1 vs. L3:** A statistical significant difference for perception threshold at [100 750] Hz is shown in Table 2 with L1 having a lower PT.

**L2 vs. L3:** No statistical significant difference was obtained for perception threshold at [100 750] Hz shown in Table 2.

Note that the *p*-values are at 0.059 for both cases, therefore they are only slightly above significance level of *p* < 0.05.

#### 3.1.2. Just noticeable difference

**Figure 4:**
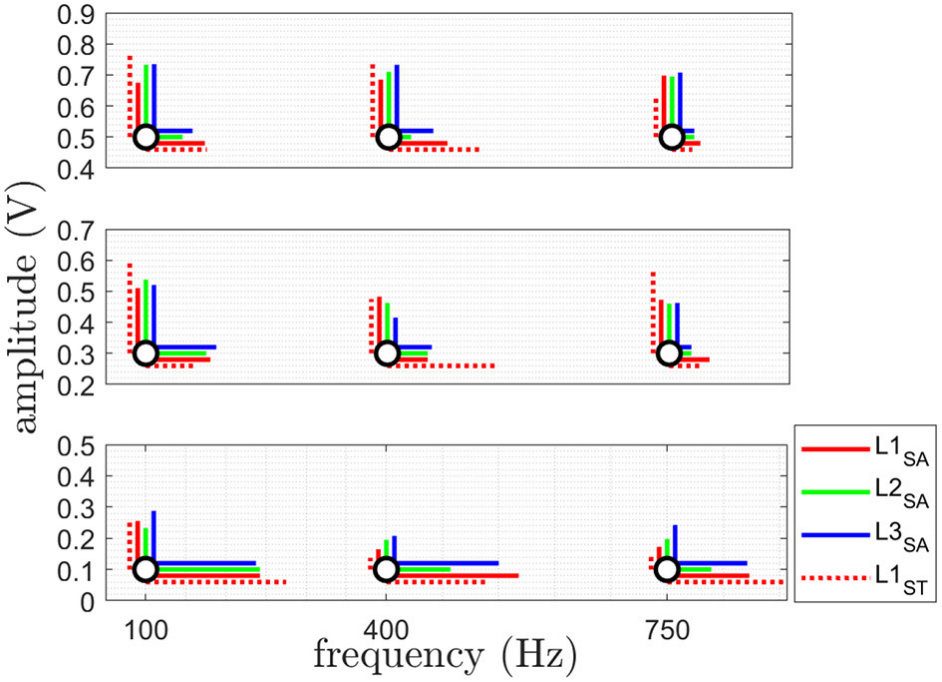
This plot shows the combination of JND_*a*_ (vertically) and JND_*f*_ (horizontally) at each AF point. The JND_*a*_ showing the amplitude resolution for 3 different frequencies at 3 different amplitudes and JND_*f*_ giving the frequency resolution for 3 different amplitudes at 3 different frequencies in a summary plot of the obtained mean value of JND_*a*_ (blue) and JND_*f*_ (red) at each reference stimulus (black) giving. See Figure 5 for the detailed trends of each JND_*a*_ and JND_*f*_. The results are shown for all three sites L1-L3 for the able-bodied subjects (SA) and for site L1 subjects with trans-radial amputation (ST).

**Figure 5:**
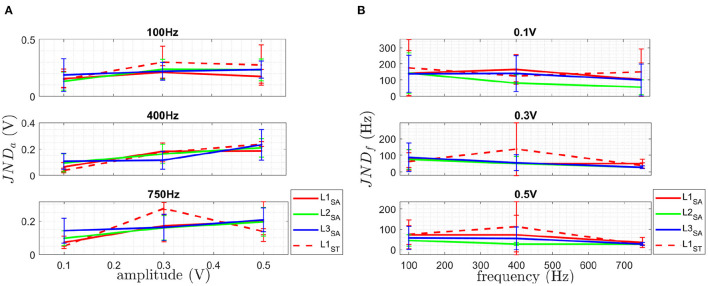
The plot in **(A)** shows the detailed JND_*a*_ at the three different chosen AF reference frequencies for the three different AF reference amplitudes and **(B)** shows the detailed JND_*f*_ for at the three different chosen AF reference amplitudes for the three different AF reference frequencies. Both are given for the three sites L1-L3 for *SA* and *ST*. The mean is plotted as solid line and the standard deviation given as error bar.

The results for the just noticable difference in amplitude JND_*a*_ are shown in **Figure 5A** and Table 3. The results for the just noticable difference in frequency JND_*f*_ are shown in **Figure 5B** and Table 4. A combined plot is shown in Figure 4, showing the mean at each site. The details of JND_*a*_ and JND_*f*_ are shown and discussed in the following.

**Table 3:** The *p*-values of the Friedman for JND_*a*_ comparing the three different physiological sites L1, L2, and L3 at all frequencies and amplitudes of the ten able-bodied subjects (SA) is given.

		**Amplitude**	**Frequency**		
			**100 Hz**	**400 Hz**	**750 Hz**
Friedman	L1 vs. L2 vs. L3	*0.1 V*	0.318	0.063	0.255
		0.3 V	0.900	0.407	0.905
		0.5 V	0.509	0.527	0.318

Significance level is taken at *p* < 0.05.

**Table 4:** The *p*-values of the Friedman for JND_*f*_ comparing the three different physiological sites L1, L2, and L3 at all frequencies and amplitudes is given.

		**Amplitude**	**Frequency**		
			**100 Hz**	**400 Hz**	**750 Hz**
Friedman	L1 vs. L2 vs. L3	0.1 V	0.828	0.018*	0.174
		0.3 V	0.565	0.717	0.008*
		0.5 V	0.140	0.143	0.500
Wilcoxon	***L*1*vs*.*L*2**	0.1 V	-	0.031*	-
		0.3 V	-	-	0.031*
	***L*1*vs*.*L*3**	0.1 V	-	0.406	-
		0.3 V	-	-	0.063
	***L*2*vs*.*L*3**	0.1 V	-	0.094	-
		0.3 V	-	-	1.0

Significance level is *p* < 0.05. Furthermore, the *p*-values of the *post-hoc* Wilcoxon signed rank test for JND_*f*_ comparing the three different physiological sites L1, L2 and L3 to each other over all frequencies and amplitudes of the ten able-bodied subjects is shown. Significance level is *p* < 0.05. *Indicates statistically significance.

##### 3.1.2.1. JND_*a*_

The obtained results of JND_*a*_ are shown in Figure 5A. The obtained results of the Friedman test comparing the three sites, as shown in Table 3, indicate no statistically significant difference between the three physiological sites, therefore no *post-hoc* test was performed.

##### 3.1.2.2. JND_*f*_

The obtained results of JND_*f*_ are shown in Figure 5B. The results of the Friedman test for the three sites of JND_*f*_, as shown in Table 4, indicate a statistically significant difference for JND_*f*_ at [0.1 V, 400 Hz] as well as for [0.3 V, 750 Hz]. No statistically significant difference was found for all other discrete steps in the AF domain. The results of the *post-hoc* Wilcoxon signed rank test comparing the three different physiological sites L1, L2 and L3 to each other for 0.1 V at 400 Hz and 0.3 V at 750 Hz, shown in Table 4. In the following, the obtained results are summarized:

**L1 vs. L2:** A statistical significant difference for JND_*f*_ at 400 Hz for 0.1 V and 0.3 V is shown in Table 4.

No statistical significant difference was obtained for 0.1 V at 400 Hz and 0.3 V at 750 Hz is shown for

**L1 vs. L3** as well as **L2 vs. L3** in Table 4.

#### 3.1.3. Single-point location identification rate

**Figure 6:**
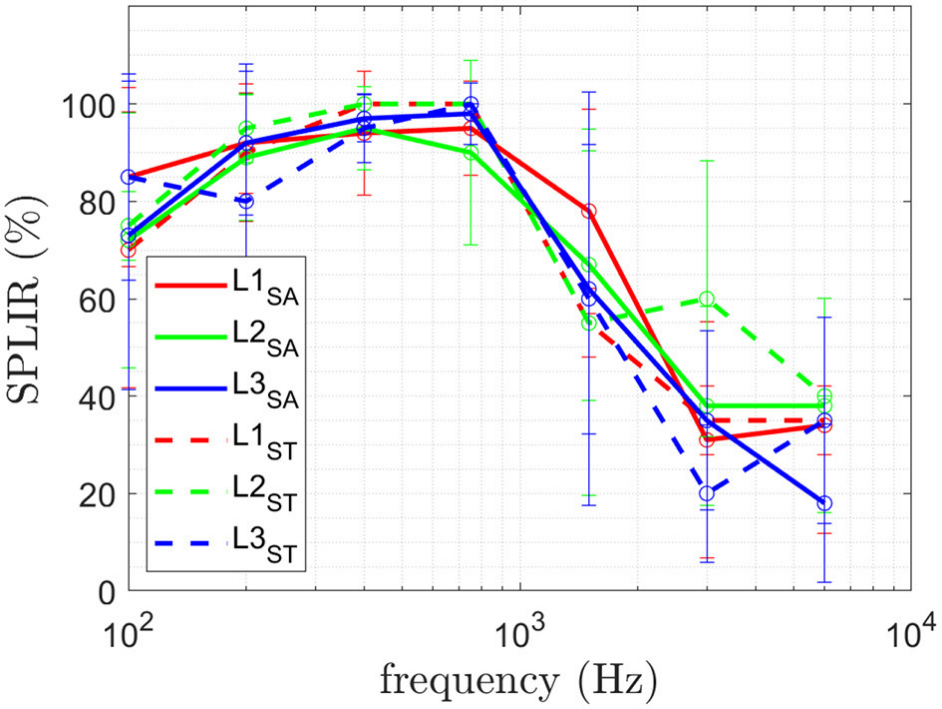
The mean and standard deviation of the SPLIR for each site L1, L2, and L3 is shown. The results of the ten able-bodied subjects (SA) is plotted in solid lines and for subjects with trans-radial amputation (ST).

The results of the obtained SPLIR are shown in Figure 6 for all three physiological sites. The Friedman results for the three different sites, as shown in Table 5, indicate no statistically significant difference in performance for the different sites for SPLIR, hence, no *post-hoc* test was performed.

**Table 5:** The *p*-values of the Friedman for SPLIR comparing the three different physiological sites L1, L2, and L3 at all frequencies and amplitudes is given.

	**Frequency**						
	**100 Hz**	**200 Hz**	**400 Hz**	**750 Hz**	**1,500 Hz**	**3,000 Hz**	**6,000 Hz**
L1 vs. L2 vs. L3	0.581	0.460	0.867	0.104	0.081	0.704	0.103

Significance level is *p* < 0.05. *Indicates statistically significance.

### 3.2. Subject groups

The obtained perception threshold of the two user groups, able-bodied subjects (SA) and subjects with trans-radial amputation (ST), are shown in Figure 3. The results show a lower to similar perception threshold for *ST* compared to *SA*. A higher value is obtained for L3 at 750 Hz.

The obtained JND_*a*_ of the two user groups is shown in Figure 5A. The obtained JND_*f*_ of the two user groups is shown in Figure 5B. The obtained SPLIR of the two user groups is shown in Figure 6.

## 4. Discussion

In this section, the obtained results are discussed for: (a) the three physiological sites; (b) the different subject groups and compared to results obtained for invasive bone conduction in Clemente et al. (2017).

### 4.1. Physiological sites

For perception threshold site L1 performed better (in statistically significance manner) than L2 and L3 at 100 and 750 Hz, while no difference was obtained at 400 Hz. Better performance of L1 can be explained by on the one hand allowing better contact to the bones in a non-invasive manner due to little skin and soft tissue in between transducer and bone. On the other hand the ulnar nerve runs behind the medial epicondyle on the inside of the elbow and might increase the perception by being mechanically stimulated. The results of the study did not reveal any statistically significant differences in PT between the three stimulation sites at 400 Hz. While the L1 site demonstrated the lowest value, the high variance of the results could account for the lack of statistical significance (see Figure 3). One possible explanation for the variability in the results is the limited number of participants in the study. Another factor to consider is the findings of Clemente et al. (2017), which suggest that stimulation frequencies above 400 Hz can be perceived as sound. As a result, the change in sensation from tactile-only to tactile and audio could contribute to the increased variability observed in the results. The obtained PT achieved for invasive bone conduction (Clemente et al., 2017) which is a mean PT of [0.2, 0.1, 0.47] N compared to the results obtained in this study being [0.01, 0.45, 0.20] N for *SA* and [0.01, 0.21, 0.25] N for *ST* at f=[100, 400, 750] Hz. A smaller perception threshold at 100 Hz and 750 Hz for all three sites has been achieved for both user groups for non-invasive bone conduction feedback. The obtained PT at 400 Hz is higher than in Clemente et al. (2017) for all three sites.

Note that a lower perception threshold is the desired performance. Performing better with respect to PT means a lower perception threshold was obtained, allowing to use a bigger force range and therefore a larger bandwidth for the feedback information is available. It also allows the use of smaller transducers, as investigated in Mayer et al. (2020a), and therefore reduced energy consumption for battery powered prosthesis. Furthermore, lower stimulation force reduces the resulting noise and therefore increases the likelihood of the acceptance within the prosthetic field.

For JND_*a*_, no statistically significant difference between different sites at the three different frequencies was obtained. As a result, each of the three sites is equally sensitive.

For JND_*f*_, a statistically significant difference between different sites at [0.1 V, 400 Hz] as well as for [0.3 V, 750 Hz] was found. No statistical difference was found for all other stimulation permutations. In combination with the significantly lower PT, L1 has the biggest bandwidth for providing sensory feedback.

For SPLIR, no statistically significant difference between the different sites at the different frequencies was obtained, meaning each stimulation on each site can be located equally well. As reported previously in preliminary studies in Mayer et al. (2020c), SPLIR drops to at chance level above 1,500 Hz. Such a drop suggests that the site identification is superior for tactile perception, which is prevalent below 750 Hz (Mayer et al., 2019), and the stimulation location can not be perceived auditory perception. The stimulation in the case of auditory perception is conducted *via* the bones to the auditory pathway. Stimulating on three different independent sites on the elbow will ultimately still lead to stimulation of the same auditory pathway and hence not allow the subject to distinguish the stimulation site.

### 4.2. Subject groups

Only qualitative discussion is performed in the comparison of subject groups, due to the small number of ST subjects available for the study. For perception threshold, subjects with trans-radial amputation qualitative show a lower PT than able-bodied subjects and therefore are more sensitive to stimulation. This allows the use of a wider force range / larger bandwidth for the feedback information, and the use of smaller transducers for reduced energy consumption. Furthermore, the subjects with trans-radial amputation achieve similar performance for PT using non invasive bone conduction compared to the subjects with invasive bone conduction studied in Clemente et al. (2017). For JND_*a*_, similar to PT a slightly better performance was obtained for *ST* compared to *SA*. A smaller JND_*a*_ means a higher resolution of the feedback interface is feasible and therefore more detailed sensory feedback can be provided.

For JND_*f*_, a slightly worse performance was obtained for *ST* compared to *SA*. A higher JND_*f*_ means a coarser resolution of the feedback interface necessary and therefore less detailed sensory feedback can be provided.

For SPLIR, similar performance for *ST* compared to *SA* was obtained. Both subject groups SPLIR drops to at chance level above 1,500 Hz, suggesting that the site identification is superior for tactile perception for both subject groups.

#### 4.2.1. Established interface parameters

The results obtained in this study suggest a usable bandwidth for bone conduction as a sensory feedback from 100 to 750 Hz when multiple transducers are used on multiple stimulation sites. The perception threshold is as low as 0.01 N at 100 Hz and increases to 0.2 N at 750 Hz. This range of force perception and frequency bandwidth suggest that commercially available transducers used in audiology could be used for bone conduction sensory feedback. Furthermore, the perception threshold for non-invasive bone conduction was found to be comparable to results obtained with invasive bone conduction techniques (Clemente et al., 2017). The study also revealed that frequency resolution was more distinguishable than amplitude. This finding could have implications for the design of future bone conduction feedback systems, as it suggests that greater attention may need to be paid to the frequency content of the feedback signals.

It should be mentioned that sensory feedback bone conduction is not applicable for subjects with diseases affecting the perception of such stimulation e.g., a potential subject in this study had to be excluded due to the inability to perceive stimulation likely caused by rheumatoid arthritis.

## 5. Conclusion

This study has evaluated the temporal and spatial parameters of the non-invasive vibrotactile feedback on the bony landmarks of the elbow. The parameters are investigated on three different physiological sites over two user groups (able bodies and subjects with transradial amputation). The paper reports the effective operating range of frequencies and amplitudes and the resolutions that can be perceived generally by the human users.

The perception threshold on the ulnar olecranon (L1) is most sensitive for able-bodied subjects compared to the medial and lateral epicondylus. The perception threshold is lower and therefore more sensitive for subjects with trans-radial amputation compared to able-bodied subjects. A qualitatively smaller, and therefore more sensitive, perception threshold has been obtained compared to invasive bone conduction.

Previous research (Clemente et al., 2017) showed that osseoperception, caused by mechanical vibrations through a bone-anchored (osseointegrated) prostheses, allows for a richer feedback and therefore was believed to play an important role in the sense of ownership of a prosthesis and the improvement of quality of live of people living with limb loss. The equivalent sensitivity achieved in non-invasive bone-conduction within this study highlights the potential of such an interface for conventional socket-based prostheses to not only provide richer feedback and functionality but also to enhance the sense of ownership of a prosthesis.

The resolution in amplitude and frequency of all three sites, as well as for able-bodied subject vs. subjects with trans-radial amputation, showed comparable performance. The detection of the stimulation site was not different between different sites as well as the two investigated user groups.

## Data availability statement

The raw data supporting the conclusions of this article will be made available by the authors, without undue reservation.

## Ethics statement

The studies involving human participants were reviewed and approved by Engineering Human Ethics Advisory Group at the University of Melbourne. The patients/participants provided their written informed consent to participate in this study.

## Author contributions

RM, AM, YT, and DO: literature, experiment, data, analysis, and paper. GA and PC: paper design, experiment design, and paper review. All authors contributed to the article and approved the submitted version.
